# Artificial Intelligence and Machine Learning Technologies in Cancer Care: Addressing Disparities, Bias, and Data Diversity

**DOI:** 10.1158/2159-8290.CD-22-0373

**Published:** 2022-06-02

**Authors:** Irene Dankwa-Mullan, Dilhan Weeraratne

**Affiliations:** IBM Watson Health, IBM Corporation, Cambridge, Massachusetts.

## Abstract

Artificial intelligence (AI) and machine learning (ML) technologies have not only tremendous potential to augment clinical decision-making and enhance quality care and precision medicine efforts, but also the potential to worsen existing health disparities without a thoughtful, transparent, and inclusive approach that includes addressing bias in their design and implementation along the cancer discovery and care continuum. We discuss applications of AI/ML tools in cancer and provide recommendations for addressing and mitigating potential bias with AI and ML technologies while promoting cancer health equity.

## INTRODUCTION

Advances in artificial intelligence (AI) and machine learning (ML) technologies hold promise for personalized, equitable cancer care and improved health outcomes. The potential of the tools to generate insights from massive amounts of data, in ways that can help inform decisions, interventions, and precision cancer care, is enormous. Opportunities include being able to inform personalized care, improve early detection and screening methods, and derive insights from multidimensional data sets. Other aspects include analysis of multiomics data for diagnostic, prognostic, and therapeutic markers, interpretation of radiology and histopathology images, clinical trials, and preclinical research including drug discovery. Advances in precision oncology have been tied to the generation of genomic data and a deeper understanding of tumor biology and progression through AI-enabled technologies. As the technology matures and data accumulate at a massive scale, AI and ML will continue to play a critical role in optimizing administrative and clinical efficiency to enable personalized precision cancer care through rich clinical, genomic, and social determinants of data. An important aspect will be addressing disparities in early detection, screening, treatment, survivorship, and quality of life for patients experiencing social disadvantage and barriers to cancer care. There are growing concerns that these technologies may further exacerbate disparities in cancer care because of the lack of affordability, inconsistent accessibility, and biased AI and ML models. Identifying and understanding concerns and addressing sources of bias contributing to the disparities will help formulate successful approaches to adequately optimize the potential of AI–ML to promote equity in cancer care. This article will discuss the use of AI and ML in cancer care and provide a framework for mitigating bias to achieve cancer equity leveraging the potential of these technologies.

## APPLICATIONS OF AI AND ML IN CANCER

The advent of multiomics technology including genomics, proteomics, transcriptomics, and metabolomics has been revolutionary in cancer diagnosis, prognosis, and treatment. However, the increasing complexity and volume of omics data have unveiled new opportunities to use AI–ML methods to make meaningful clinical associations. ML approaches, including supervised, unsupervised, and reinforcement learning, have been used to integrate and analyze multiomics data to predict early detection, recurrence, prognosis, and risk stratification and subtyping in cancer. Additionally, ML approaches have been developed to reduce multidimensionality in omics data to predict success to chemotherapy, targeted therapy, and immunotherapy (1). Finally, ML algorithms have been developed to integrate multiomics data with radiology and digital pathology data to augment decisions on prognostic biomarkers differentiating radiation-sensitive and radiation-resistant tumors and draw more composite inferences (2).

AI use in tumor histopathology and radiology imaging runs the gamut including early detection, accurate diagnosis, subtyping, determining stage and grade, and predicting prognosis. A random forest classifier has been used in the early detection of eight different types of cancers using a simple blood test. Convoluted neural networks (CNN) have been used to distinguish malignant tumors from benign lesions in breast, colorectal, and gastric cancers with imaging slides (3). A CNN-based model has been used to subtype lung tumors into small cell carcinoma, adenocarcinoma, and squamous cell carcinoma. ML algorithms have been used to differentiate low grade versus high grade in colorectal and prostate cancers. A deep learning algorithm developed to detect lymph node metastasis in breast cancer performed better than a panel of 11 pathologists (3). Deep learning algorithms that assess histology slides have been used to predict prognosis and clinical outcome in colorectal cancer and glioblastoma. In addition, a logistic regression–based method to generate classifier-trained genomic profiles was used to predict resistance to immunotherapy in patients with advanced melanoma (4). Finally, ML methods can be used to identify genetic lesions from histopathology slides obviating the need for wet lab assays such as immunohistochemistry (IHC), fluorescent *in situ* hybridization, or next-generation sequencing.

AI has been used in different facets of preclinical and clinical research, including target identification, drug discovery, drug design, and repurposing and synergy. A deep learning classification approach has been used to predict novel drug targets associated with breast cancer pathogenesis (5). Similar algorithms have been used to predict drug efficacy and synergy using cancer cell line data. An ML model has been trained to predict absorption, distribution, metabolism, and excretion (ADME) properties of new drugs. Patient transcriptomics and genomics profiles have been used to create algorithms to repurpose drugs for bladder cancer (5).

Deep learning algorithms have been used to optimize clinical workflows, including in histopathology where it is used to automate quantification and classification of cell types. These algorithms are integrated into clinical workflows for mutation prediction for prescreening and definitive testing in many cancer types (6).

ML applications have also emerged as pivotal in cancer care delivery processes and clinical decision support systems (CDSS). Algorithms focusing on text mining from electronic health records (EHR) and clinical practice guidelines and automated extraction of concepts have been fundamental to developing an effective CDSS. Such AI-based CDSSs have been reported to significantly increase diagnostic accuracy and clinical guideline adherence and reduce physician burden (7).

## REALIZING THE PROMISE OF AI AND ML IN CANCER CARE

ML and AI can substantially improve health care delivery. However, ML models are usually built on historical data, and consequently groups that have been historically sidelined, or experienced barriers to care, can be affected by data as well as analytic and algorithmic biases. These groups include racial and ethnic population groups, socioeconomically disadvantaged populations, and sexual orientation and gender identity groups. Racial biases can unintentionally skew health care predictive models and marginalize protected groups. Algorithms developed to predict clinical outcome will be inherently biased if the training set is not racially and demographically representative.

A gaping underrepresentation of enrollment of African Americans and older individuals has historically been reported in clinical trials. Advances in deep learning are facilitating the planning and execution while ensuring diversity in trials. ML models can mitigate recruitment bias by optimizing patient cohort selection and inclusion criteria. In addition, AI is used to enrich biomarker-driven patient cohorts in basket and umbrella trials. Mutation prediction ML systems that analyze histopathology slides can be used to screen large multiracial patient cohorts inexpensively to aid patient stratification in biomarker-based cancer trials. The ML analysis of the histopathology slides can also be used to predict treatment response in trials (8).

Cancer care is inexorably moving toward genomics-driven treatment, which argues for reduced morbidity and mortality. However, there are conspicuous inequities in the whole-exome and whole-genome profiling of patients of different races. A predominant majority of sequenced patients in The Cancer Genome Atlas project are of European ancestry, with underrepresentation of Asians, Africans, and Hispanics ancestry. ML models that are trained on racially skewed sequencing data may not holistically represent prognostic, diagnostic, and therapeutic genetic signatures across races. For example, the incidence of *FOXA1* mutations in prostate cancer was significantly higher, whereas *TP53* mutations were significantly lower in black men compared with white men. Similarly, a higher frequency of mutations in familial cancer susceptibility genes has been attributed to the higher incidence of prostate cancer in African-American men (9). Black women with hormone receptor–positive breast cancer have worse prognosis compared with their white counterparts. As such, genetic profiles of diverse populations should be incorporated into any ML algorithms that abet the execution and delivery of personalized medicine to patients with cancer to improve model accuracy.

## OPPORTUNITIES FOR ADDRESSING BIAS IN AI AND ML MODELS TO PROMOTE CANCER HEALTH EQUITY

Bias is human and deeply entrenched in our society. It is hard to avoid bias. Health care decisions stemming from trials that are not diverse or inclusive or data evidence that is not aligned with the reality of a majority of patients in racially diverse and socially disadvantaged communities produce unreliable results. We have a responsibility to ensure that our data, practice, and science evidence that informs these tools augments decision-making for everyone without further disadvantaging or discriminating other groups. Therefore, building equitable, transparent, and fair AI and ML systems in health should be an urgent priority.

To ensure equity in developing and deploying ML algorithms in health care, Rajkomar and colleagues have proposed the use of principles of distributive justice (10). ML models in health care should be developed such that protected and nonprotected demography groups derive equal clinical benefit, calibrated to perform equally between the groups and finally to ensure equitable resource allocation and demographic parity during deployment. During the evaluation phase of the algorithm, model performance should be assessed across different patient population groups. In addition, historical data on which the model is predicated should be assessed to determine whether these data would amplify and perpetuate racial bias. After deployment of an algorithm, performance metric should be monitored across groups to ensure equal performance. The ML models’ infinite capacity to continue training as more data are available should be availed to further refine algorithms. Our recent published work outlines a proposed framework for ethical AI in ways that integrate health equity and social justice principles in the AI development life cycle (11). Inclusive data and evidence generation as well as bias mitigation in AI and ML technologies are going to become increasingly critical for delivering high-quality and equitable cancer care.

## RECOMMENDATIONS FOR ADDRESSING BIAS AND ACHIEVING CANCER EQUITY

**Figure 1.**
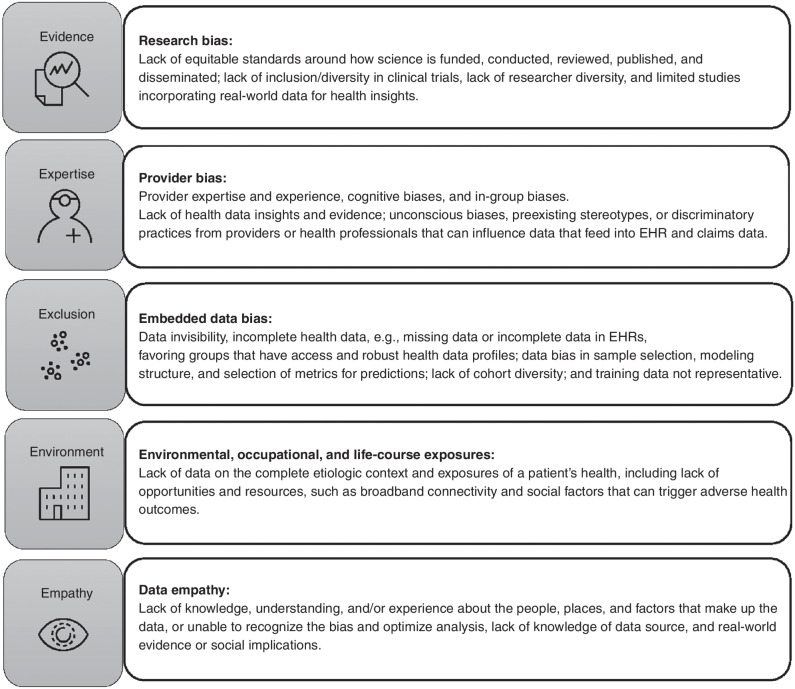
The five major sources of bias across the data generation and implementation cycle for AI and ML systems, where patterns are analyzed, insights are extracted, decisions are made, and ultimately action is taken on the data. Strategies for addressing bias are structured around the sources of bias and include (i) evidence or research bias, (ii) expertise or provider bias, (iii) exclusion or embedded data bias, (iv) environmental or life-course exposures, and (v) empathy or bias in appropriately contextualizing data.

Data sources for informing clinical evidence, developing cancer care guidelines, and building training algorithms to inform management decisions and resource allocation largely come from research trials, EHRs, and administrative claims data. Data almost always have a human being in the loop, deciding on how the data are generated, supported, and collected and how the evidence is translated into practice. As a result, bias is introduced not only into the training data sets that generate the algorithms but also in the process of evidence generation and establishing standards for the models. Thus, addressing deeply embedded bias to optimize our AI–ML tools requires a multi-stakeholder strategic and collaborative approach. We present a proposed strategy for identifying bias and recommendations that will guide the development of promoting equitable cancer care leveraging AI–ML technologies. The recommendation is structured around the sources of bias in AI and ML, shown in Fig. 1, which includes (i) evidence or research bias, (ii) expertise or provider bias, (iii) exclusion or embedded data bias, (iv) environmental or life-course exposures, and (v) empathy or bias in appropriately contextualizing data.

## STRATEGIES FOR ADDRESSING BIAS

### Prioritize Inclusive Research Trials and Data Diversity for Building an Evidence Base

Clinical management for cancer prevention, early detection, screening, and treatment is informed by synthesis of evidence that is tied to clinical trials, a rigorous scientific randomized control trial, or real-world evidence studies. Recommendations for evidence-based clinical decision-making are based on a synthesis of evidence studies, including randomized controlled clinical trials, real-world evidence, and practice-based guidelines from consensus panels. It is well-known that the current state of clinical trials or scientific studies does not always match the demographics of the patient population that is at risk or suffers from the disease condition being studied. Despite a bold 5-year plan by the FDA to improve diversity and transparency in clinical trials for newly approved drugs, black patients have remained inadequately represented and, of these trials, fewer than 20% met the requirement of reporting on race-specific benefits and adverse effects (12). Our research and data generation efforts for building the evidence base and context for clinical decision-making that informs medical AI and ML algorithms should be grounded in equity and inclusion. Incorporating real-world data and other relevant sources of health data helps provide greater clarity and add value to AI–ML algorithms.

### Address Unconscious Biases in Cancer Care

Health provider expertise and practice delivery are an integral part of generating and translating evidence into cancer clinical care for improved health and outcomes. Data that are transcribed into EHR systems for patient care, administrative claims, and cancer databases inform predictive, prognostic, and risk algorithms in cancer care. A patient may arrive at a medical facility with symptoms. The primary care practitioner will determine the course of action based on their assessment and beliefs, often in combination with tumor biomarker, lab results, or imaging data. They may combine sources of patient-generated data with professional guidelines including on evidence-based practice to make treatment decisions. The cancer care management and treatment plan can be based on a thorough examination of the patient, listening to their story, and understanding their values, preferences, culture, beliefs, and life experiences. But the actions can also include unconscious (or conscious) bias, stemming either from experience with a cohort of similar patients or from their own stereotypes or other influences. Unconscious bias may rely on stereotypes and can lead to discriminatory practices. Actions influenced by compassion or values for equity and fairness can help drive and ensure patient satisfaction and optimal health outcomes. Addressing unconscious bias and promoting patient-centered care are important aspects for promoting AI–ML fairness, optimizing algorithmic bias, and achieving cancer health equity. Trainings on cultural competency and algorithmic vigilance, including awareness of our own human biases, can help address this critical source of bias.

### Establish Standards in Data Collection and Generation

Bias can occur with inappropriate data standards or lack of comprehensive and accurate collection and generation of sociodemographic data. This includes lack of diversity and exclusion of relevant information such as patients’ needs, values, preferences, and life-course experience and history and how these determinants shape their risk for cancer health outcomes. Bias can also be introduced where there is a lack of consistent race and ethnicity data collection or adequate standards to capture the diversity and unique experiences of different patient groups. Promoting equity includes establishing appropriate categories of race, ethnicity, gender, or disability status, including workplace and the range of those determinants of health that we know have been linked to health. We have an increasingly diverse patient population with unique social factors. Our current research practices around data reinforce norms of homogeneity for racial and ethnic minority populations. We apply similar standards and aggregate our statistical comparisons for groups such as Black people, Asian American communities, those of Hispanic ethnicity, Indigenous people, even though members are heterogeneous with varied environmental and social exposures as well as different risk attributes that may contribute to differential outcomes. Accurate standards and collection of detailed demographic data could optimize AI–ML algorithms to produce more accurate predictions.

### Integrate Relevant Social Determinants of Health Data in AI–ML Algorithms

Social determinants of health are those structural determinants and conditions in which people are born, grow, live, work, and age. These determinants include socioeconomic status, neighborhood social capital, and the physical environment, education, food, community social support networks as well as access to health care (13). Capturing data on environmental, occupational, and life-course exposures and relevant social determinants of health is important to understand factors that may influence cancer risk and outcomes. Medical care alone cannot address what makes us sick. Our AI/ML research and algorithm development should seek to integrate the complete etiologic “context” of a patient's health to advance cancer care. We can optimize AI and ML models by including relevant social indicators or predictors of health. Building such tools will also require including and building trust with communities and population groups.

### Promote Data Empathy in Cancer Care

Data empathy refers to how much empathy, patient values and preferences, reported experiences, and reported outcomes are integrated into care and decision-making. It includes developing an understanding about the experiences, culture, and factors that influence health behaviors about the people, the places, or the factors that made up the data. Lack of empathy of the sources and context of data results in an inability to optimize the algorithm or the decision-making process. Health data need to be foundational enough to be exchangeable globally across multiple platforms, but human enough to convey the story, the values, and experiences of patients and populations.

## CONCLUSION

AI and ML technologies have significantly shaped many aspects of cancer care in recent years. Addressing the multiple sources of embedded bias to optimize these tools for cancer health equity requires a systemic, coordinated, and collaborative approach. Determinants of health and disease are multifactorial and complex, and our AI–ML technologies should reflect this complexity. Promoting health equity requires our humanity, our empathy, and transparency in our data generation and AI–ML implementation efforts. In addition, we need to work collaboratively across multiple stakeholders and especially with all communities to inform rich data standards that integrate social determinants and a framework for generating responsible data and unbiased ethical and trustworthy AI–ML technologies.
